# Population insight of the relationship between lifestyle and cancer: A population-based survey

**DOI:** 10.3934/publichealth.2019.1.34

**Published:** 2019-01-23

**Authors:** Fawaz Dabea Alshammari, Hussain Gadelkarim Ahmed, Dena Alshammari, Ahmed Mulfy Alharbi, Atif Saud Alsaedi, Abdulbaset Elasbaly

**Affiliations:** 1 Department of Clinical Laboratory, College of Applied Medical Science, University of Hail, Kingdom of Saudi Arabia (KSA); 2 Department of Pathology, College of Medicine, Molecular Diagnostics and Personalized Therapeutics Unit, University of Hail, KSA; 3 Department of Clinical Laboratory Sciences, College of Applied Medical sciences, Jouf University, Skaka, KSA

**Keywords:** cancer awareness, lifestyle, Saudi Arabia, cancer, risk factors

## Abstract

**Background:**

There is a substantial rise in the incidence of cancer in Saudi Arabia. Life style models and lack of awareness are the prime suspect in this substantial increase. Therefore, the objective of the present study was to assess the relationship between lifestyle and cancer in a population-based Survey in Northern Saudi Arabia.

**Methodology:**

This cross-sectional study was conducted in North Saudi Arabia (Hail Region). Data was collected as a part of a community based cancer's awareness movement that covered an area inhibited with approximately 500,000 individuals.

**Results:**

In this study, about 2558/3227 (79.3%) and 641/794 (80.7%) believed that tobacco smoking and smokeless are not a risk of cancer development. In this study large section (87.2%) of the study population believe that exposure to diverse occupational or non-occupational chemicals has no role in cancer development. Furthermore, around 59% of the study subjects in the current study believed that repeated exposure to insecticidal chemicals doesn't influence the risk of cancer.

**Conclusion:**

The present study point to the urgent need for awareness educational programs and preventive measures towards may lifestyle factors that can increase or decrease the overall risk of cancer among Saudi population.

## 1. Introduction

The global burden of cancer continues to increase largely both in developed and developing countries [1]. The incidence of cancer continues to rise every years due to accumulation of several risk factors including; increasing tobacco use, physical inactivity, overweight, aging and shifting reproductive patterns accompanying urbanization and economic growth. In 2012, there were 14.1 million new cancer cases and 8.2 million deaths worldwide. In recent years it was observed that the burden of cancer has shifted toward developing world, which represented about 57% of new cases and 65% of cancer deaths worldwide [2]. The incidence of the new cancer cases in developing countries is expected to increase from about 56% to more than 60% of the world's total in 2030, which is attributed to the increasing trends in cancer rates and anticipated growths in life expectancy and growth of the population [3].

However, implementing systematic, equitable and evidence-based strategies for prevention, early detection, diagnosis, treatment and palliation using available resources is a program known as national cancer control program (NCCP) designed by World Health Organization (WHO) to reduce the morbidity and mortality of cancer and improve quality of life of cancer patients. Regardless of resource limitations a country experiencing, when well-perceived and well-managed, a NCCP supports decrease the cancer burden and improve facilities for cancer patients and their families [4].

Increased weight or obesity elevates the risk of several cancers in many organs including; esophagus, colorectum, breast endometrium and kidney. It is necessary to maintain the body mass index within the normal range (18.5–25 kg/m^2^). Regular physical activity, decreases the risk of colorectal cancer and breast cancer. Alcoholic beverage consumption was found to increase the risk of some cancers including; liver, esophagus, pharynx, oral cavity and breast cancer. Foods frequently contaminated with Aflatoxin increases the risk of hepatocellular carcinoma. Salt preserved foods and high salt consumption perhaps upsurge the risk of carcinoma of the stomach. Certain salted fish was found to increase the risk of nasopharyngeal carcinoma. Preserved food particularly red meat possibly increase the risk for colorectal cancer.

Regular intake of Fruits and vegetables most likely decrease the risk of several cancers including; the cancers of oral cavity, esophagus, stomach and colorectum [5].

There is a substantial rise in the incidence of some cancers in Saudi Arabia in recent years due to several etiological risk factors, which differ for different geographical regions in the country [6]. Life style models and lack of awareness are the most common factors expected to contribute to substantial increase in the incidence of cancer in Saudi Arabia [7]–[11]. Increase of age in general population as well as, obesity are the most challenging factors contributing to the etiology of cancer in Saudi Arabia [12].

Cancer awareness is an increasingly important issue in light of increasing incidence and associated healthcare costs, as well as the presence of risk management strategies [13]. However, the great majority of cancers arise as a consequence of modifiable life style risk factors; therefore community education program targeting cancer risk factors is crucial to reduce the overall incidence of cancer [14].

Therefore, the objective of the present study was to assess the relationship between lifestyle and cancer: A population-based Survey in Saudi Arabia.

## 2. Materials and methods

This cross-sectional study was conducted in North Saudi Arabia (Hail Region). Data was collected as a part of a community based cancer's awareness movement that covered an area inhibited with approximately 500,000 individuals. The sample size was calculated using online sample size calculator available at: https://www.calculator.net/sample-size-calculator.html.

Applying confidence interval of 60 ± 1.66 and confidence level of 95%, the sample size was 3326.

Participants were targeted in different public settings including University of Hail. Each participant was asked to fill a questionnaire about tobacco and alcohol habits and other information regarding their attitudes towards these factors in relation to cancer etiology. Beside the demographical characteristics, the questionnaire also included; Living with smoker, Smokeless tobacco; Chemical usage, Insecticidal exposure, Vegetable and fruit wash before eating, Radiation exposure, Soft or refined food can increase the risk of cancer, Preserved food can increase the risk of cancer, Some food can increase the risk of cancer, Some food can decrease the risk of cancer, Natural food can decrease the risk of cancer, Vegetable and fruit intake decrease cancer risk, Green Tea and other antioxidant decrease cancer risk, Physical activity decrease cancer risk, Increased body weight increases cancer risk, Obesity can increase the risk of cancer.

### 2.1. Statistical analysis

Statistical analysis was performed by proportion. The Microsoft Excel Office 2007 and the SPSS software (version 16) were used for statistical analysis. The software was used for calculation and production of frequencies and percentages.

### 2.2. Ethical consent

Written informed consent was obtained from each respondent, ensuring strict anonymity. The Ethical Committee of the Department of Pathology, College of Medicine at the University of Hail has approved the study.

## 3. Results

This study included 3326 participants, their ages ranging from 15 to 77 years old with a mean age of 25 years. Out of 3253 respondents for gender classification, 1701 (52.3%) were males and 1552 (47.7%) were females.

**Figure 1.**
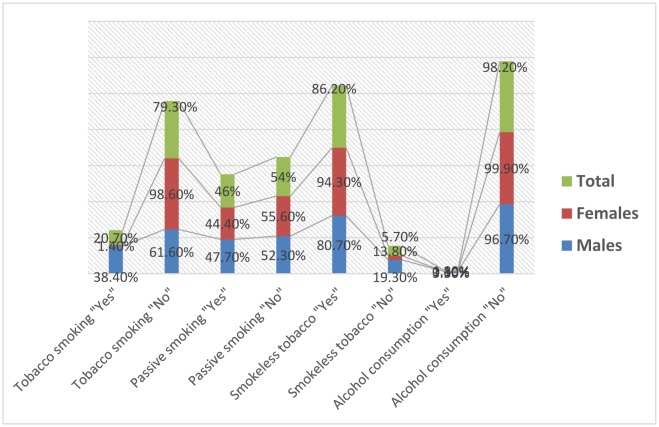
The proportions of the relationship between cancer, tobacco exposure, and alcohol consumption.

The knowledge about the relationship between cancer risk and factors such as tobacco usage and alcoholic beverages consumption was summarized in Table 1. Out of 3227 respondents, 669/3227 (20.7%) participants were found to believe that tobacco smoking is a major risk factors for cancer and the remaining 2558/3227 (79.3%) had indicated that tobacco smoking is not risk factors. Out of the 669 answered “yes”, 647/1684 (38.4%) were males compared to 22/1543 (1.4%) females. Out of 2558 participants answered “No”, 1037/1684 (61.6%) were males and 1521/1543 (98.6%) were females, as indicated in Table 1, Figure 1.

With regard to exposure to passive smoking (living with smoker), 1430/3103 (46%) participants were found to believe that exposure to passive smoke is a risk for cancer and the remaining 1673/3103 (54%), were not. Out of 1430 respondents “Yes”, 761/1597 (47.7%) were males and 669/1506 (44.4%) were females. Out of 1673 respondent “No”, 836/1597 (52.3%) were males and 837/1506 (55.6%) were females, as shown in Table 1, Figure 1.

With regard to smokeless tobacco, 1139/1322 (86.2%) participants has pointed out that, smokeless tobacco is a major risk for cancer and only 183/1322 (13.8%) have answered “No”. Out of the 1139 “Yes” respondents, 641/794 (80.7%) were males and 498/528 (94.3%) were females. Out of 183 answered “No”, 153/794 (19.3%) were males and 30/528 (5.7%) were females, as shown in Table 1, Figure 1.

For alcoholic beverages consumption, only 57/3207 (1.8%) have agreed that alcohol consumption may be a risk factor for cancer and the remaining 3150/3207 (98.2%) believed that alcohol beverage consumption is not considered as a cancer risk factor, as indicated in Table 1, Figure 1.

**Table 1.** The relationship between cancer, tobacco exposure, and alcohol consumption.

Variable	Category	Males	Females	Total
*Tobacco smoking*				
	Yes	647	22	669
	No	1037	1521	2558
	Total	1684	1543	3227
*Living with smoker*				
	Yes	761	669	1430
	No	836	837	1673
	Total	1597	1506	3103
*Smokeless tobacco*				
	Yes	641	498	1139
	No	153	30	183
	Total	794	528	1322
*Alcohol consumption*				
	Yes	55	2	57
	No	1607	1543	3150
	Total	1662	1545	3207

**Figure 2.**
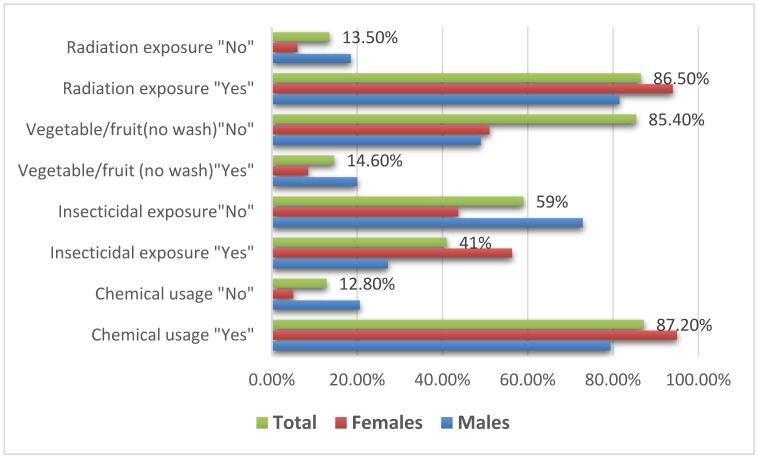
The relationship between cancer, chemical usage, insecticidal, and radiation exposure.

The knowledge on the relationship between cancer, chemical usage, insecticidal, and radiation exposure was summarized in Table 2, Figure 2. Out of the 1884 respondents, about 1640/1880 (87.2%) participants believed that comprehensive repeated chemical substances usage is a major cancer risk factor and the remaining 240/1880 (12.8%) have reverse believe. Out of the 240 participants answered “No”, 194/944 (20.6%) were males and the remaining 46/936 (5%) were females.

Out of the 3153 respondents, about 1292/3153 (41%) participants believed that comprehensive repeated exposure to insecticidal chemicals is a major cancer risk factor and the remaining 1861/3153 (59%) have reverse believe. Out of the 1861 participants answered “No”, 1210/1663 (72.9%) were males and the remaining 651/1490 (43.7%) were females.

Out of the 3207 respondents, about 469/3207 (14.6%) participants believed that comprehensive repeated eating of vegetables and fruits without wash is a major cancer risk factor and the remaining 2738/3207 (85.4%) have reverse believe. Out of the 2738 participants answered “No”, 1341/2738 (49%) were males and the remaining 1397/2738 (51%) were females.

Out of the 1750 respondents, about 1514/1750 (86.5%) participants believed that exposure to radiation is a major cancer risk factor and the remaining 236/1750 (13.5%) have reverse believe. Out of the 236 participants answered “No”, 195/1055 (18.5%) were males and the remaining 41/695 (6%) were females, as indicated in Table 2, Figure 2.

**Table 2.** The relationship between cancer, chemical usage, insecticidal, and radiation exposure.

Variable	Category	Males	Females	Total
*Chemical usage*				
	Yes	750	890	1640
	No	194	46	240
	Total	944	936	1880
*Insecticidal exposure*				
	Yes	453	839	1292
	No	1210	651	1861
	Total	1663	1490	3153
*Vegetable and fruit wash before eating*				
	Yes	339	130	469
	No	1341	1397	2738
	Total	1680	1527	3207
*Radiation exposure*				
	Yes	860	654	1514
	No	195	41	236
	Total	1055	695	1750

**Figure 3.**
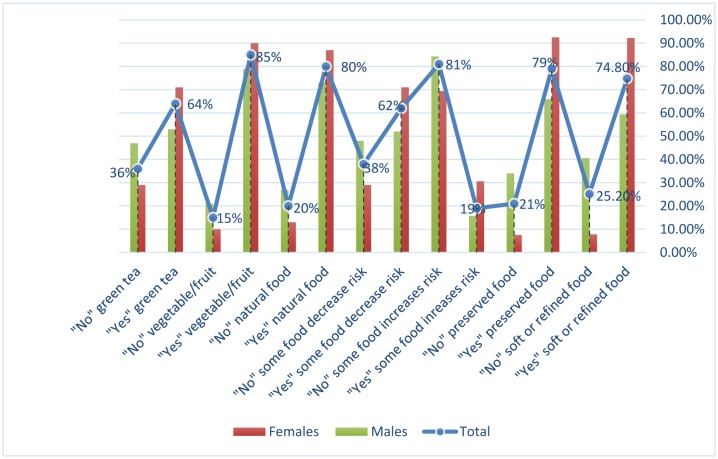
The relationship between cancer, and food habits.

The relationship between cancer, and food habits was summarized in Table 3, Figure 3. When asking the participants whether soft or refined food can increase the risk of cancer, around 1230 participants have responded. Out 1230 respondents, 920 (74.8%) (388 were males and 532 were females) answered “Yes” and 310 (25.2%) (265 were males and 45 were females) answered “No”.

When asking the participants whether preserved food can increase the risk of cancer, about 1372 participants have responded. Out 1372 respondents, 1082 (79%) (462 were males and 620 were females) answered “Yes” and 290 (21%) (240 were males and 50 were females) answered “No”.

When asking the participants whether some food can increase the risk of cancer, about 465 participants have responded. Out 465 respondents, 89 (19%) (56 were males and 33 were females) answered “Yes” and 376 (81%) (301 were males and 75 were females) answered “No”.

When asking the participants whether some food can decrease the risk of cancer, about 2858 participants have responded. Out 2858 respondents, 1767 (19%) (708 were males and 1059 were females) answered “Yes” and 1091 (81%) (653 were males and 438 were females) answered “No”.

When asking the participants whether natural food can decrease the risk of cancer, about 2581 participants have responded. Out 2581 respondents, 2072 (80%) (873 were males and 1199 were females) answered “Yes” and 509 (20%) (328 were males and 181 were females) answered “No”.

When asking the participants whether vegetable/fruit intake can decrease the risk of cancer, about 2039 participants have responded. Out 2039 respondents, 1738 (80%) (654 were males and 1084 were females) answered “Yes” and 301 (20%) (177 were males and 124 were females) answered “No”.

When asking the participants whether anti-oxidants such as green tea intake can decrease the risk of cancer, about 1834 participants have responded. Out 1834 respondents, 1169 (64%) (393 were males and 776 were females) answered “Yes” and 301 (36%) (345 were males and 320 were females) answered “No”, as shown in Table 3, Figure 3.

**Table 3.** The relationship between cancer, and food habits.

Variable	Category	Males	Females	Total
*Soft or refined food can increase the risk of cancer*				
	Yes	388	532	920
	No	265	45	310
	Total	653	577	1230
*Preserved food can increase the risk of cancer*				
	Yes	462	620	1082
	No	240	50	290
	Total	702	670	1372
*Some food can increase the risk of cancer*				
	Yes	56	33	89
	No	301	75	376
	Total	357	108	465
*Some food can decrease the risk of cancer*				
	Yes	708	1059	1767
	No	653	438	1091
	Total	1361	1497	2858
*Natural food can decrease the risk of cancer*				
	Yes	873	1199	2072
	No	328	181	509
	Total	1201	1380	2581
*Vegetable and fruit intake decrease cancer risk*				
	Yes	654	1084	1738
	No	177	124	301
	Total	831	1208	2039
*Green Tea and other antioxidant decrease cancer risk*				
	Yes	393	776	1169
	No	345	320	665
	Total	738	1096	1834

**Figure 4.**
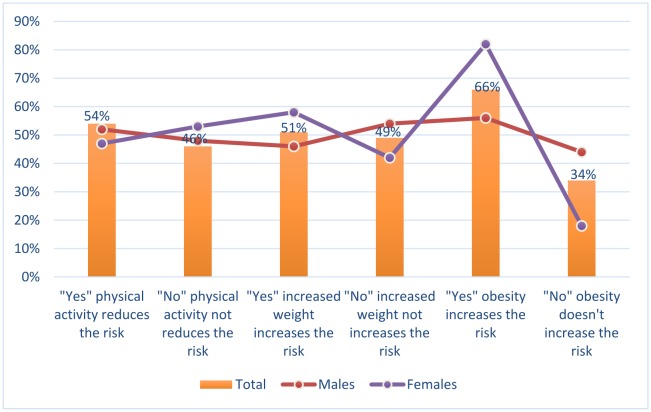
The relationship between cancer, and body weight and physical activity.

The relationship between cancer, and body weight and physical activity was summarized in Table 4, Figure 4. With regard to the physical activity as a factor that can decrease the risk of cancer, out of 3193 respondents, 1711 (54%) participants greed “Yes” (874 were males and 837 were females) that physical activity can reduce the risk of cancer, hence, about 1482 (46%) participants (793 were males and 898 were females) disagreed “No”.

With regard to increased body weight as a factor that can increase the risk of cancer, out of 3172 respondents, 1631 (51%) participants greed “Yes” (759 were males and 872 were females) that increased body weight can increase the risk of cancer, hence, about 1541 (49%) participants (898 were males and 643 were females) disagreed “No”.

With regard to obesity as a factor that can increase the risk of cancer, out of 1124 respondents, 745 (66%) participants greed “Yes” (379 were males and 366 were females) that obesity can increase the risk of cancer, hence, about 379 (34%) participants (299 were males and 80 were females) disagreed “No”.

**Table 4.** The relationship between cancer, and body weight and physical activity.

Variable	Category	Males	Females	Total
*Physical activity decrease cancer risk*				
	Yes	874	837	1711
	No	793	689	1482
	Total	1667	1526	3193
*Increased body weight increases cancer risk*				
	Yes	759	872	1631
	No	898	643	1541
	Total	1657	1515	3172
*Obesity can increase the risk of cancer*				
	Yes	379	366	745
	No	299	80	379
	Total	678	446	1124

## 4. Discussion

Cancer represent a major health problem worldwide. Although the prevalence of cancer is high in developed countries, but there is a tremendous increase of cancer in developing countries. In light of increasing of new cancer cases, there is an uprising need for implementing cancer awareness programs.

In the present study we tried to highlight some important cancer issues in order to find out the gaps in this context to make available more information to take effective cancer prevention strategies.

In the current study we screened a large population in a cross sectional survey in Hail region (Northern Saudi Arabia) in order to evaluate the level community awareness and knowledge toward some cancer related life style habits. Hail is a province of Saudi Arabia, located in the north of the country. It has an area of 103,887 km^2^ and a population of 547226 (2010 census) [15]. Hail's people are social able and they have the custom of regular gathering in what is known locally as “Diwania”, therefore, is always opportunities to spread the awareness through these public gathers.

In this study, about 79.3% and 80.7% believed that tobacco smoking and smokeless are not a risk of cancer development. These findings have great discrepancies from other studies that reported much lower percentages. In study to evaluate the awareness of oral cancer and perception of tobacco use cessation counseling 97.6% Indian, 96.5% Saudi, 96.5% Yemeni and 98.4% United Arab Emirates respondents recognized the association between oral cancer and cigarette smoking [16]. Another study indicated that tobacco chewing (84%), tobacco chewing with areca nut (68%), chewing areca nut alone (51%) and exposure to actinic radiation (71%) as risk factors [17]. Another study from Yemen indicted that about 71.5% of the participants had heard about oral cancer. Smoking and smokeless tobacco usage were identified as the major risk factors by 71.5% and 73.7% of the participants, respectively [18]. In a study to assess the level of awareness regarding association between tobacco use and cancer, the risk awareness of the smoking lung cancer link was 83.6%, while the risk awareness of the smoking heart disease link was 46.5% [19]. In a study from Saudi Arabia to assess the level of awareness and knowledge about signs and risk factors of oral cancer in the general population in Saudi Arabia. Only 53.6% of the participants had heard of oral cancer. Smoking and alcohol consumption were identified as the major risk factors by 81.7% and 56.3% of the participants, respectively [20]. However, the acute differences in these studies compared to our findings might be attributed to study population. The majority of these previous studies devoted to students and more educated population settings, hence, our study was restricted to deep community base setting in northern Saudi Arabia. The level of education and less exposure to civilization in addition to the lack of awareness might strongly contribute the findings of the present study.

In the present study about 98.2% of the participants disagreed that alcohol consumption is a risk factor for cancer development. Although it was well established that alcohol beverages consumption is a risk for the development of several precancerous and cancerous lesions [21]–[23], but there is a lack of data in this regard from Saudi Arabia. However, studies from Saudi Arabia have showed low epidemiological values in this regard [24],[25]. In Saudi Arabia, alcohol beverage consumption is illegal and also considered as social stigma, thus it is difficult to find the exact epidemiological measures.

In this study large section (87.2%) of the study population believe that exposure to diverse occupational or non-occupational chemicals has no role in cancer development. The exposure to diverse types of chemical substances has been linked to a number of cancers. Early-life arsenic exposure, has been linked to lung and bladder cancer [26], workplace chemical exposures (soldering materials) has been linked to breast cancer [27]. Occupational exposure to diverse chemical in industrialized areas has been linked to several cancers [28]. However the only study in this context from Saudi Arabia linked the occupational benzene exposure in petroleum stations to development of bladder precancerous changes [29].

Furthermore, around 59% of the study subject in the current study believed that repeated exposure to insecticidal chemicals doesn't influence the risk of cancer. Exposure to insecticidal chemicals such as Organochlorine insecticides has been linked to some cancers, particularly leukemia [30],[31].

In the present study about 85.4% of the participants believed that comprehensive repeated eating of vegetables and fruits without wash doesn't increase the risk of cancer. The majority of these vegetables and fruits are contaminated with various chemicals including pesticides insecticides, and herbicides, the risk of which is well established [30],[32],[33].

With regard to the exposure to radiation, approximately 86.5% of the participants believed that exposure to radiation is a major cancer risk factor. This is high percentage of awareness toward radiation hazard.

When asked the participants whether soft or refined food can increase the risk of cancer, around 74.8% answered yes. Many studies have suggested the relationship between intake of refined and fast food and the risk of cancer [34]–[36], which requires, the undertaking of certain educational and preventive measures.

When asked the participants whether preserved food can increase the risk of cancer, about 79% of the participants agreed. However, the intake of preserved foods was positively associated with the incidence of epithelial ovarian cancer [37], as well as, esophageal carcinoma [38].

On we asked the participants whether some food can increase the risk of cancer, only 19% have answered yes. It was suggested that vegetable fiber and total fiber play very important roles in protecting against colorectal cancer [39]. Increased intake of omega-3 fatty acids associated with decreased omega-6—resulting in higher omega-3 to omega-6 ratio compared with Western-type diet—is inversely associated with breast cancer risk [40]. Moreover, similar results were found on asked the participants whether some food can decrease the risk of cancer. There are several dietary types can reduces the risk of some cancers. It was found that soy intake possibly decreases the risk of breast cancer [41].

On asking the participants whether natural food can decrease the risk of cancer, 80% answered yes. Because of their role as antioxidants, the intake of carotenoids has been found to reduce the risk of head and neck cancer (HNC) [42]. The consumption of fruits is well known to reduce the risk of human cancers. Since oxidative stress and chronic inflammation play important roles in cancer development, dried fruits with antioxidative and anti-inflammatory properties hold promise for cancer chemoprevention. The antioxidant, anti-inflammatory and chemopreventive activities of dried fruits are largely attributed to their polyphenols and vitamins. Dried fruits contain adequate amounts of bioactive principles, such as anthocyanins, acetogenins, catechins, coumarins, phenolic acids, terpenes, xanthones, and others [43].

On asking the participants whether vegetable/fruit intake can decrease the risk of cancer, about 80% agreed. A higher consumption of vegetable and fruit was found to be associated with a decreased risk of several cancers, such as breast cancer, hepatocellular carcinoma, gastric carcinoma etc [44]–[46].

When asking the participants whether anti-oxidants such as green tea intake can decrease the risk of cancer, about 64% agreed. An inverse association between green tea intake and lung cancer risk has been observed among never smokers but not among smokers [47]. There are insufficient evidences to support green tea consumption reduces the risk of esophageal cancer, gastric cancer, and pancreatic cancer [48]–[50].

With regard to the physical activity as a factor that can decrease the risk of cancer, about 46% of the study population disagreed. Physical activity is consistently associated with a reduced risk of colorectal cancer in epidemiologic studies. Overall, a stronger relative risk of physical activity on colorectal cancer risk was observed in the higher body mass index group, although the difference was not statistically significant, suggesting an added benefit of physical activity as a cancer prevention strategy in population groups with strong risk factors for colorectal cancer [51]. Exercise promotes significant improvements in clinical, functional, and in some populations, survival outcomes and can be recommended regardless of the type of cancer. Although generally safe, patients should be screened and appropriate precautions taken. Efforts to strengthen uniformity in clinical trial reporting, develop clinical practice guidelines, and integrate exercise and rehabilitation services into the cancer delivery system are needed [52].

With regard to increased body weight as a factor that can increase the risk of cancer, about 49% of the study population in the present study disagreed. he joint association between energy balance and cancer risk are hypothesized to share the same underlying mechanisms, the amplification of chemical mediators that modulate cancer risk depending on the responsiveness to those hormones to the target tissue of interest. Disentangling the connection between obesity, the insulin-IGF axis, endogenous hormones, inflammatory markers, and their molecular interaction is vital [53]. The association between obesity and several cancers is well-established [54],[55]. Alcohol consumption, salt consumption, red meat consumption and Aflatoxin contamination are considered as cancer risks [56].

The limitations in the current study include its cross-sectional design. Longitudinal studies in the future might give up insight into developments in the outcomes of existing and future lifestyle related cancer control measures. Another limitation of the present study is that data were obtained via self-report.

## 5. Conclusions

The present study point to the urgent need for awareness educational programs and preventive measures towards may lifestyle factors that can increase or decrease the overall risk of cancer among Saudi population. There is a necessity for in depth cancer prevention related studies and further specific measurements to prove that life style risk factors have influence on cancer burden in Saudi Arabia and in Northern Saudi Arabia in particular.
